# Channel-spatial attention network for fewshot classification

**DOI:** 10.1371/journal.pone.0225426

**Published:** 2019-12-12

**Authors:** Yan Zhang, Min Fang, Nian Wang

**Affiliations:** School of Electronics and Information Engineering, Anhui University, Hefei, China; Hangzhou Normal University, CHINA

## Abstract

Learning a powerful representation for a class with few labeled samples is a challenging problem. Although some state-of-the-art few-shot learning algorithms perform well based on meta-learning, they only focus on novel network architecture and fail to take advantage of the knowledge of every classification task. In this paper, to accomplish this goal, it proposes to combine the channel attention and spatial attention module (C-SAM), the C-SAM can mine deeply more effective information using samples of different classes that exist in different tasks. The residual network is used to alleviate the loss of the underlying semantic information when the network is deeper. Finally, a relation network including a C-SAM is applied to act as a classifier, which avoids learning more redundant information and compares the relation between difference samples. The experiment was carried out using the proposed method on six datasets, such as *mini*imagenet, Omniglot, Caltech-UCSD Birds, describable textures dataset, Stanford Dogs and Stanford Cars. The experimental results show that the C-SAM outperforms many state-of-the-art few-shot classification methods.

## Introduction

### Background

Recently, many state-of-the-art deep learning algorithms with different classification problem have been proposed [1, 2]. These algorithms have achieved better performance when a large amount of data is available. In the real world, enough labeled samples of many classes are hard to obtain. For example, newly discovered *birds*, newly designed *cars* etc. If these categories with a few samples are classified with traditional deep learning algorithms, the results maybe overfit and the model cannot directly transfer to other new classification tasks. Conversely, humans can find a similar image effortlessly when one or several images are shown to them, because humans have largely learned prior knowledge from experience [3]. When the model has enough meta-knowledge, it will solve many different tasks without a large number of labeled samples.

Motivated by the performance of human, some state-of-the-art one- or few-shot algorithms based on meta-learning have been proposed [4, 5], where the meta-learning aims to learn a distribution for a new task from many past different tasks, instead of learning the representation of the classes. For example, the Model-Agnostic Meta-Learning for Fast Adaptation of Deep Networks [6] (MAML) trained a meta-learner by gradually changing the gradient to converge on many tasks, so that the meta-learner has good initial parameters to adapt quickly to the new task, but it takes some time to update a few steps before it can perform well on testing set. Metric-based algorithm [7, 8] is another key method that attempts to compute distance in the embedding space. In this direction, Snell et al. [9] applied convolutional neural network to extract features of samples, and applied the Euclidean distance to compute the similarity of the features of different samples. Although the above methods perform well, these results of the methods can be further improved. The core problem of few-shot classification is to solve empirical risk minimization [10]. This reason is that we only get several training samples, this makes the empirical risk deviates from the optimal result. To solve it, few-shot classification methods mainly are divided into two strategies: data augmentation and prior knowledge. Data augmentation aims to augment the number of training samples, but this method causes the robustness of the few-shot classification model is poor when the training samples are less. Such as data rotation, data crop and so on. Prior knowledge aims to transfer related knowledge to target task from a series of available tasks. In other word, few-shot classification need consider that whether similar tasks are always sufficient. Moreover, it is challenge for few-shot classification to transfer knowledge for target task from the same task but different domains. To this end, this paper aims to further dig related information existing in different datasets based on prior knowledge.

In recent years, the attention mechanism [11] has attracted widely interest for extracting abundant features in computer vision systems. Humans can quickly recognize objects by focusing on certain areas of the object when the object is seen, because the human visual system can pay attention to salient parts. There are two types of attention mechanism: channel attention and spatial attention. The channel attention focuses on global features given some feature maps, while the spatial attention focuses on local features given a feature map. The channel attention mechanism [12] produces one dimension (1D) tensor for given feature maps, which is activated by the sigmoid function. In a few channel axes of feature maps, some activation values of the 1D tensor are expected to higher over the corresponding feature maps of interest and some activation values are expected to lower to reduce the redundant feature maps. The spatial attention mechanism [13] is vital to focus on salient parts, which produces a feature mask that has the same size as same as the given feature maps, but the channel attention mask has not the same size as same as the given feature maps. In a network, the spatial mask is expected to automatically adjust activation values, some corresponding parts of interest are focused over feature maps when the activation values are higher.

In fact, the different classes existing in different tasks contain a large number of related features in few-shot classification, and further knowledge of which can be transferred to new classes. This paper combines the two types of attention mechanisms to extract abundant features of the image across many historical tasks. The reasons as follows: firstly, this channel attention focuses on feature maps of interest, there are a lot of information is ignored in the feature maps when lower activation values are multiplied by the corresponding feature map. Secondly, the spatial attention focuses on some parts of interest over the feature maps. When the channel attention weaken the information existing in some feature maps, the spatial attention can emphasize a great deal of useful parts of every feature map with the attention mask in another branch. Finally, the output feature maps of two attention mechanisms are fused by addition operation. These features of interest are richer and those redundant features are cut in fused feature maps. Some state-of-the-art methods perform surprisingly using the relation module [14, 15] instead of the distance metric, because the relation module with the learning parameter can avoid irrelevant information. Therefore, this paper also uses a relation module.

The main contributions of this paper are as follows:

1. This paper combines the spatial attention and the channel attention. In particular, they are placed in different convolution layers to mine discriminative information from multi-scale feature maps.
2. A residual network is applied to aggregate the output feature maps of the C-SAM and the input feature maps of the C-SAM. The residual network can avoid the loss of underlying semantic information. The underlying semantic information is the feature information given image in first few convolution layers, and the feature maps still contain abundant information when the network is shallow.
3. This paper combines a relation module with C-SAM as a classifier. The module can measure the similarity between unlabeled and labeled images.
4. To enhance the performance of the network, this paper also chooses different customized loss functions. These loss functions are evaluated on different components of the network. Moreover, this paper also discusses the generalization on other datasets using the same trained model.
5. This proposed method and some state-of-the-art algorithms are evaluated on *mini*imageNet, Omniglot, Caltech-UCSD Birds, Stanford Dogs, Stanford Cars and describable textures dataset. The effects of different components are analyzed as well on these datasets. Experimental results show this proposed method achieves good performance from a new classification task with few labeled data.

### Related work

#### Few-shot classification

**Fig 1:**
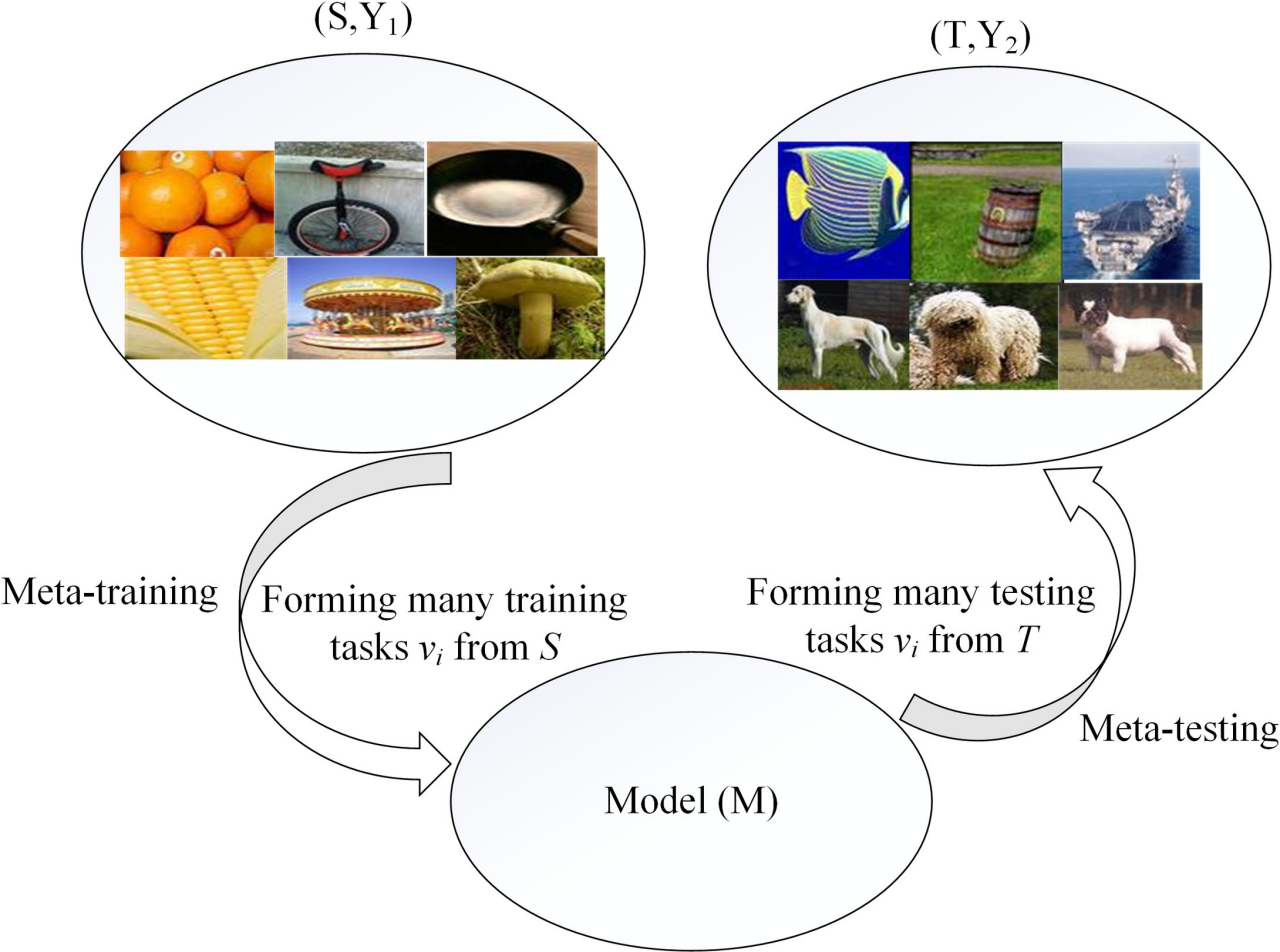
Procedure of meta-learning.

The meta-learning algorithm [16–19] is important to solve few-shot classification, because a large amount of labeled data is expensive and insufficient, which makes the generalization of many deep learning models is weak in few-shot classification. The meta-learning aims to learn a lot of meta-knowledge from a set of auxiliary tasks, which helps those models to solve novel classification tasks. Concretely, as shown in Fig 1, where *S =* {*X*_*1*_, *X*_*2*_, *X*_*3*_*…*, *X*_*s*_} represents the auxiliary dataset, *s* is the number of classes in auxiliary dataset. *X*_*s*_ is a class. *Y*_*1*_ = {*y*_*1*_, *y*_*2*_, *y*_*3*_ *…*, *y*_*s*_} is the corresponding label space and *y*_*s*_ is the label corresponding class *X*_*s*_ in *S*. *T =* {*W*_*1*_, *W2*, *W3…*, *W*_*t*_} is the target dataset, *t* is the number of classes and the *W*_*t*_ is the unseen class. *Y*_*2*_ = {*y′1*, *y′2*, *y′3…*, *y′t* } is the corresponding label space and *y′t*is the label corresponding class *W*_*t*_ in *T*. There are two stages: meta-training and meta-testing. In the meta-training stage, the task (an episode) *v*_*i*_ is defined as follows: *N* classes are randomly selected in *S* and each of the *N* classes contains *K* (*K* denotes the number of samples) samples as the training samples, the rest samples of the *N* classes as testing samples. Therefore, the model *M* is trained on a large number of tasks. In the meta-testing stage, given the new task *v*_*unseen*_ in T in the same manner, the model *M* can obtain a good generalization ability.

In this direction, some state-of-the-art few-shot learning algorithms [7–9, 14, 20] have been proposed. Compared with the MAML algorithm, metric-based few-shot learning algorithms need less time, such as the Matching networks for one shot learning [8](Matching nets) and the Prototypical networks for few-shot learning [9] (Prototypical nets). In Matching nets, the training samples and the testing samples are fed to two separate networks, an LSTM module was used to optimize the embedding function, which aims to select important information for testing samples and training samples. The similarity then is measured between the training samples and the query sample by a special metric function. The label of the query is estimated by attention mechanism over the label of the training samples. In addition, the Prototypical nets applies the Euclidean distance, which can minimize the distance between the mean of each class and the query samples. Especially, the mean of training samples of each class represents powerfully the class. Thus, the embedding network is trained to make closer a given query sample and similar labeled samples while pushing it away from unrelated labeled samples. Koch et al. [7] proposed two separate neural networks. The input pairs are inputted into the separate network simultaneously and the metric function computes the similarity between features of different samples. Above algorithms are simpler, because they only used several convolution layers. However, metric-based algorithms cannot improve a lot. The reason is that the Euclidean distance is artificially designed, and the images may be misclassified due to the redundant features. To solve this, some distance metrics need to be further improved, Boris N et al. [20] asserted that the scaled metric was important to improve the performance of few-shot classification. Because the learnable parameter on distance expression can scale distance metric, but the parameters are not easily learned when the number of training epochs is unable to determine. Moreover, Sung et al. [14] (relation net) designed a relation module as a classifier. The output of the network fed by query and the output of the same network fed by all labeled samples are cascaded. The relation module is trained to compare the relation between the labeled and the query samples. If the output is 1, it represents that two images belong to the same class, otherwise, the two images do not belong to the same class.

#### Attention mechanism

Recently, several attention mechanisms [21–23] have been used extensively in few-shot classification tasks. The attention mechanism is divided into two major styles: soft attention and hard attention. Soft attention can be considered as normalizing the weight on a neural unit, but hard attention is viewed as visible attention, which chooses an obvious region for the input images. Especially, the soft attention is used well, due to the soft attention mechanism can pay more attention to detailed information on spatial and temporal aspects. To transfer more detailed information for the new task, Qin et al. [24] added a channel attention mechanism which highlights the need to find the desired one in many feature maps. Wang et al. [25] applied a spatial attention concentrating on finding some representative receptive field in a feature map. However, many studies have only been concerned on the separate attention mechanism and two mechanisms have seldom been combined in few-shot classification. For example, matching nets [8] applied the soft attention over the labels of the training samples. This makes the label of the unseen sample is the linear combination of the labels of the seen samples.

#### Mathematical problem setting

**Fig 2:**
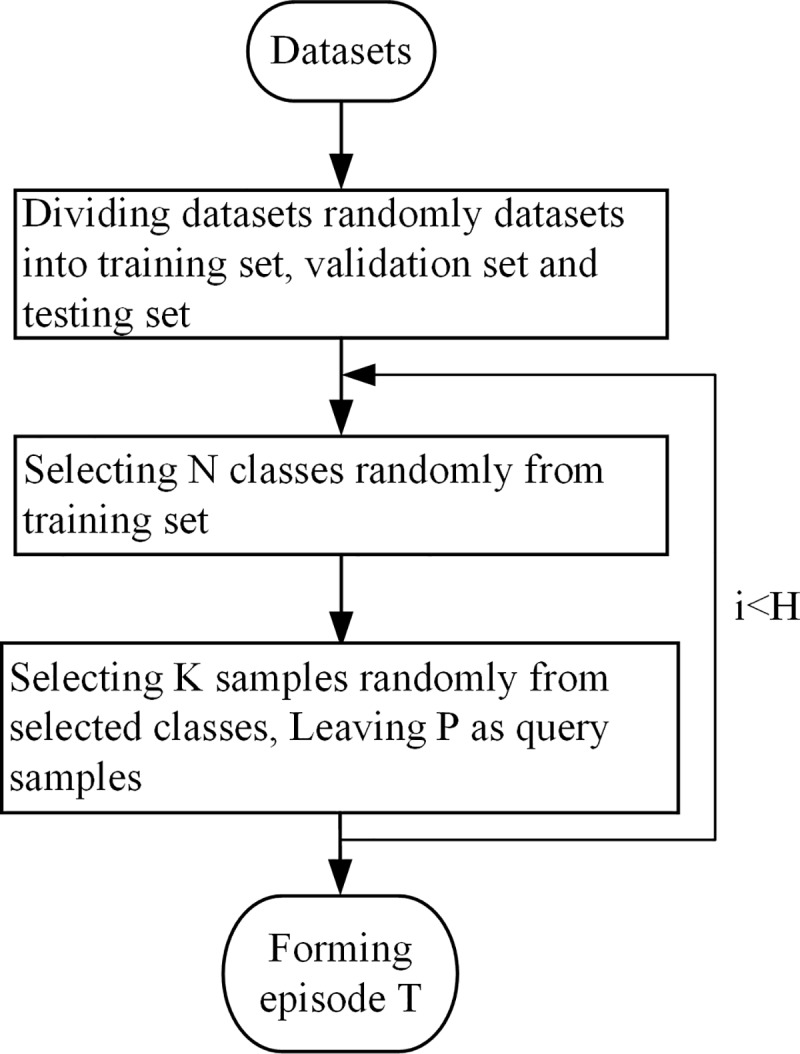
A episodic data forming.

A traditional deep neural network that requires many batch labeled images from every class. However, a large number of labeled samples cannot be easily obtained and the performance of the trained classifier is undoubtedly poor. This paper applies meta-learning to learn meta-knowledge from a support set, and the meta-knowledge can be transferred to testing set, so that the model can classify the new task with a few training samples. The support set is the training set and shares disjoint label space with testing set. Many tasks that mimic a few-shot learning setting are constructed on the training set and the testing set in the same manner. During each training procedure, the task (episode) [14] is formed as shown in Fig 2, the episode is one training data, which includes training samples and testing samples.

During training or testing, N classes (N-way) are randomly selected in the training set or testing set, and K labeled samples (K-shot) are randomly selected in each selected class as the training samples. *P* samples are randomly selected from the remaining samples as the query samples that need to be recognized. Thus, an episode (*N* way-*K* shot) is created, and H is the number of the episodes. The testing samples of an episodic data are packed into a batch, which is inputted into the network when the network starts training.

In order to compare efficiently the similarity between a few labeled and unlabeled samples, we compute the mean of a few labeled samples to represent a class in a feature space learned by a neural network. This is expressed as Eq (1), where *Q*^*N*^ *=* {*QN 1*, *QN 2 …QN k*} is a class, each *QN k* represents training samples belonging to the class *Q*^*N*^ and *k* is the number of the training samples.

$$Q^{N}=\frac{1}{k}\sum_{Q_{1}^{N}}^{Q_{k}^{N}}F_{M}\left(Q_{k}^{N};\theta_{cnn}\right)$$ (1)

## Methods

### Network architecture

**Fig 3:**
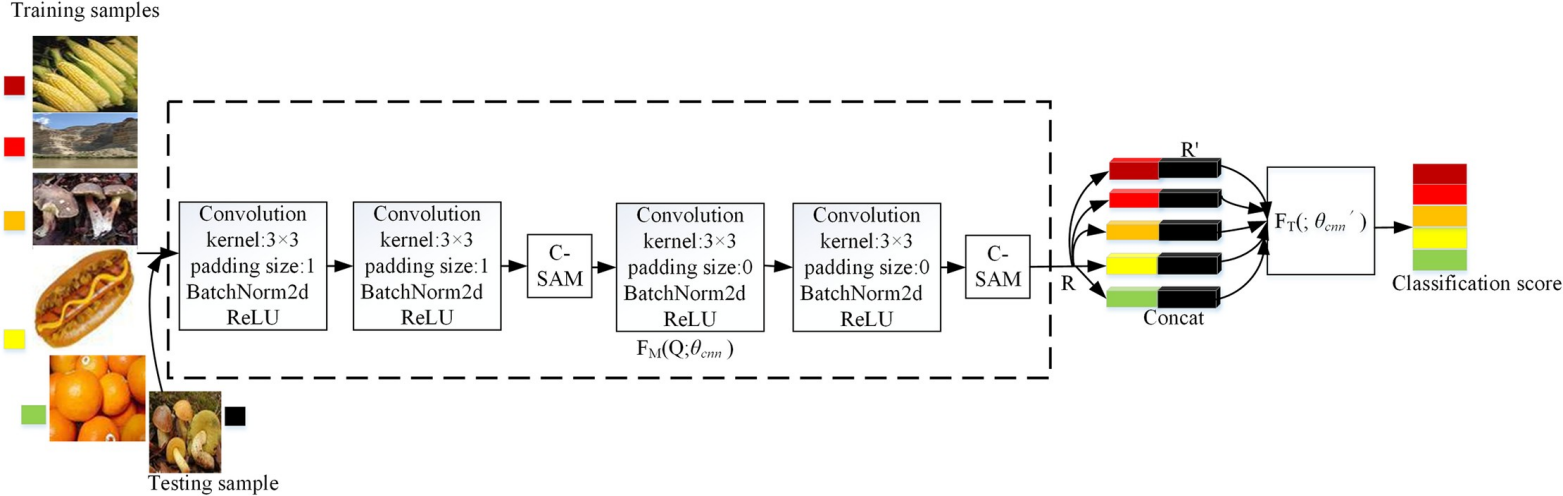
The channel-spatial attention network (C-SAM network) for 5 way-1 shot problem.

As shown in Fig 3, the model has two components: the feature extraction network *F*_*M*_ (*x*; *θ*_*cnn*_) (surrounded by the dashed line) and relation network *F*_*T*_ (; *θ*_*cnn′*_). In the feature extraction stage, if the number of parameters of the network are large, these parameters cannot be trained well, due to the lacked of samples. Finally, the network is easy to overfit. Therefore, there are four convolutional blocks and two C-SAM modules. In order to capture a large number of related information existing in different classes, the network is expected that the filter has small receptive field. Each block includes a 2D convolutional layer with a 3×3 kernel and the filter size of 64, a batch normalization layer and an activation layer. In particular, the batch normalization layer prevents gradient vanishing and speeds up the convergence. The activation layer improves the ability of the network generalization. At the same time, the two C-SAM modules are important structures of the feature extraction network, and the C-SAM are placed separately in different layers, which can further extract more detail information of each sample. In the relation network stage, there are three fully connected layers and a C-SAM module. Training sample and testing sample share the same *F*_*M*_ *(;θ*_*cnn*_*)*, while the whole flow is shown:

$$R=F_{M}(x_{i};\theta_{cnn})$$ (2)

$$r_{i,j}=sigmoid\left(F_{T}\left(concat\left(R;R^{'}\right);\theta_{cnn'}\right)\right)$$ (3)

Where *θ*_*cnn*_ is the weight of *F*_*M*_ (·), and *θ*_*cnn'*_ is the weight of *F*_*T*_(·). *x*_*i*_∈χ^C×H×W^ is the input images and χ is dimension space. R is the final feature map of training image and R ' is the feature map of testing image, and *r*_*i*,*j*_ is the relation score.

### C-SAM module

Combining the spatial attention and the channel attention have been proposed in [11–13]. Park et al. [13] proposed bottleneck attention module. Firstly, two attention masks from two separate branches are summed to form fused attention mask, the fused attention mask then multiplied by the input feature maps of the module. However, our method used two separate attention branches, and each branch gets the corresponding attention mask. Each attention mask is multiplied by the input feature maps to obtain the final output separately. Finally, the final outputs of two branches are summed by addition operation.

**Fig 4:**
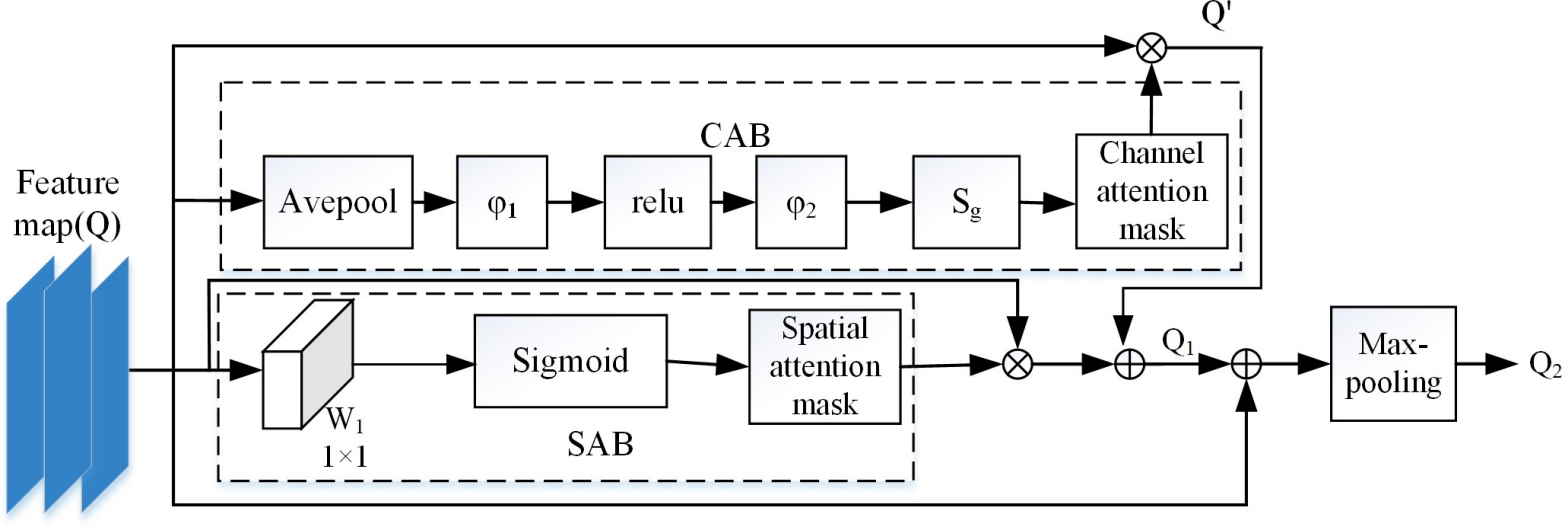
Overall architecture of the C-SAM module.

As shown in Fig 4, given an intermediate feature map *Q⊰χ*^*C×W×H*^, *C* is the number of the feature map, *W* and *H* are the width and height of the feature map respectively. After the *Q* passes the C-SAM, inferring feature map *Q'*⊰χ^*C×W×H*^ from channel attention branch and a spatial attention mask *att⊰*χ^*1×H×W*^. Fused feature maps as:

$$Q_{1}=Q⊗att⊕Q^{'}$$ (4)

In feature extraction layer, the residual network [11–13, 26] is used. Because some information of the images maybe lose when the network is deeper, which causes powerful representation of the images is difficult to be obtained, the model finally may be overfit. There are different levels of feature information existing in different layers. Thus we make the input *Q* of the C-SAM module and the output *Q*_*1*_ of the C-SAM module added to form the final feature map, following a max-pooling layer to reduce the size of the feature map. The final output *Q*_*2*_ is:

$$Q_{2}=\max\;pooling(Q⊕Q_{1})$$ (5)

### Channel attention branch

As shown in Fig 4, the channel attention branch[10, 26] (CAB) can generate the channel attention mask, namely *α*∈*χ*^*C×1×1*^, where each value of the *α* can highlight important feature maps and weaken non-essential feature maps. The *α* is obtained by Eq (6). *S*_*g*_ represents the sigmoid function [27], which can avoid excessive attenuation of useful features. The *φ_1_* and *φ_2_* are fully connected layers, *γ* is an optional parameter and the appropriate *γ* [28] can reduce the number of parameters of the model learning. Here the *γ* is set to 4. The model output is described in the following Eq (7).

$$\alpha =S_{g}\left(\varphi_{2}\left(relu\left(\varphi_{1}\left(Avepool(Q);\theta_{1}\right)\right)\right);\theta_{2}\right)$$ (6)

$$Q^{'}=\alpha ⊗Q$$ (7)

*θ*_*1*_, *θ*_*2*_ are parameters of *φ_1_*, *φ_2_* respectively. The size of *φ_1_*, *φ_2_* can be changed by adjusting γ.

### Spatial attention branch

In the previous introduction, the channel attention emphasizes which feature maps are the main ones, while the spatial attention branch [12, 13, 29] (SAB) with the dashed line in Fig 4 selects the important receptive field of the object on each feature map. Therefore, the spatial attention emphasizes a great deal of useful parts of every feature map with the attention mask in another branch when the channel attention weaken the information existing in some feature maps. The convolution layer with a 1×1 kernel namely *W*_*1*,_ which produces the original spatial attention mask *f*⊰*χ*^*1×H×W*^ as follows:

$$f=W_{1}(Q;\theta )$$ (8)

The *S*_*g*_ is used to normalize the original spatial attention mask, and *f*_*i*, *j*_ is the element of original attention mask. The *att* is computed by the following Eq (9). ⊕: represents the corresponding element of two matrixes added together, ⊗: represents the corresponding element of two matrixes multiplied together.

$$att=sigmoid(f)=\frac{1}{1+e^{-f_{i,j}}}$$ (9)

### Relation network

**Fig 5:**
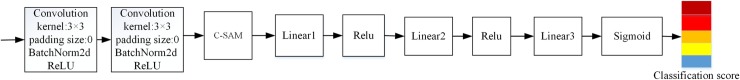
Relation module structure.

The relation module is proposed by the relation network [14], compared with the relation network, this paper adds a C-SAM branch and the fully connected layer. Firstly, the extracted feature maps of unlabeled images are concatenated with the extracted feature maps of labeled images. Then they are inputted to *F*_*T*_ (;*θ*_*cnn'*_) to learn the relationship between two samples. The module structure is shown as Fig 5. The module uses three fully connected layers of size 64, 8 and 1 respectively, and the C-SAM is placed before the fully connected layers.

The loss function is the mean squared loss function (MSE) which is expressed as Eq (10). In the formula, *r*_*i*,*j*_ represents the relationship value of the two images. When *r*_*i*,*j*_ is 1, it represents that the two images belong to the same class, otherwise, the two images do not belong to the same class. In particular, the one-hot encoding represents the corresponding label.

$$[\theta_{cnn},\theta_{cnn^{'}}]=\underset{\theta_{cnn},\theta_{cnn^{'}}}{\arg\;\min}\;\sum_{i=1}^{m}\;\sum_{j=1}^{n}\left(r_{i,j}-1(y_{i}==y_{j}\right){)}^{2}$$ (10)

## Experiment

### Experimental details

In this part, we firstly evaluate our model on three popular public datasets. These datasets are *mini*imageNet, Omniglot, and the Caltech-UCSD Birds 200 [30] (*CUB-200*). These datasets are used commonly in few-shot classification. We then evaluate our model on three novel datasets. These datasets are describable textures dataset, Stanford Dogs and Stanford Cars.

#### Public datasets in few-shot classification

The *mini*imageNet is composed of 100 categories from the ImageNet dataset, and each class contains 600 images. In this experiment, the dataset is divided into 64, 16 and 20 for training, validation and testing. The validation set aims to show visually the generalization ability of the model. At the 5 way-5 shot stage, each class has 5 labeled samples, and each class has 10 query images, and there are a total of 5×5+5×10 = 75 images in each episodic training. At the 5 way-1 shot stage, there is 1 labeled image of each class, while each class has 15 query images, and there are a total of 5×1+5×15 = 80 images in each episodic training. All input images are resized to 84×84. The Omniglot is made up of 50 different alphabets with a total of 1623 characters. 1200 classes are selected as the training set, and the remaining 423 classes are the testing set. All input images are resized to 28×28. At the 5 way-1 shot stage, each episodic training contains 5×1+5×19 = 100 images, while at the 5 way-5 shot stage, each episodic training contains 5×5+5×15 = 100 images. The *CUB-200* is fine-grained dataset that contains 200 different species of birds with a total of 11788 images. The dataset is randomly divided into 100, 50 and 50 for the training, validation and testing respectively, and follows the same episodic training principal as *mini*imagenet. The optimization uses Adam with an initial learning rate of 0.001, annealed by half for every 10^5^ episodes. Fine-tuning indicates whether the test images are used in the training process, where N means no fine-tuning with the testing set, otherwise it is Y. In the experiment, all samples of Omniglot are augmented by rotating through 90°, 180° and 270°.

**Fig 6:**
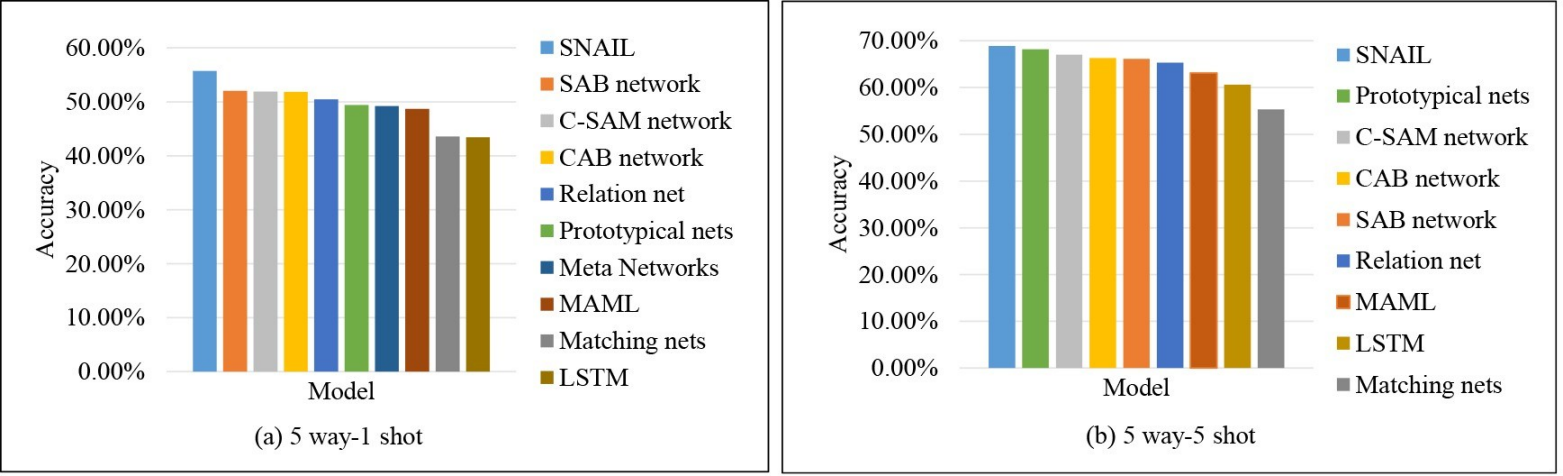
Accuracies sorted in descending order of each model on *mini*imageNet.

**Fig 7:**
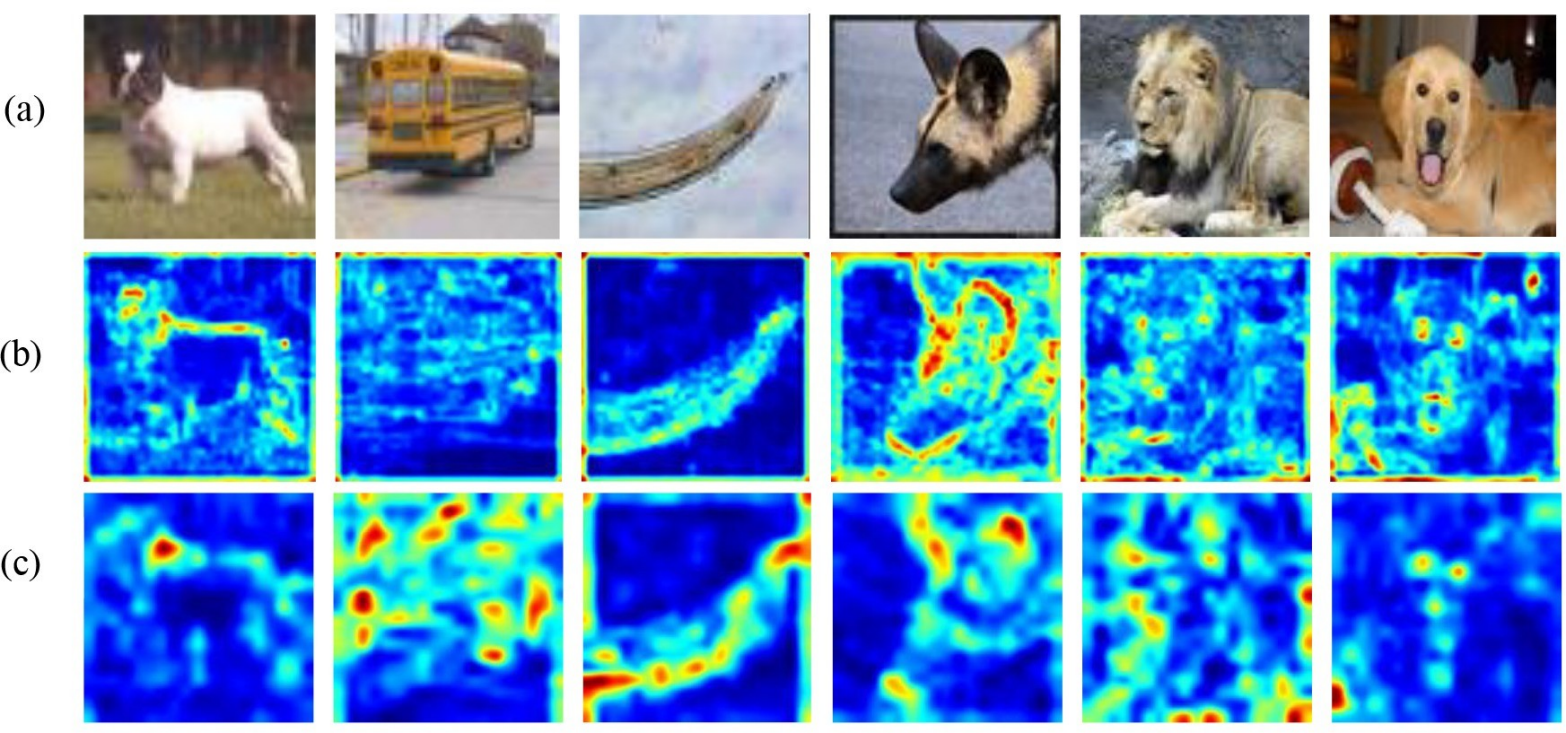
The attention map visualization of different layers on 5 way-5 shot. The input images of the proposed network. In feature extraction stage, the attention maps of different layers are visualized. (b) The attention maps come from the first C-SAM module that is added after first two convolution networks. (c) The attention maps come from the last C-SAM module. Red regions indicates some parts of the input image are more focused.

In order to prove the effectiveness of the proposed method, several state-of-the-art models are chose to compare with proposed method. As shown in Table 1, although the MAML model [6] constructed a simple network with neural network and the Optimization as a model for few-shot learning [5] (LSTM) constructed a simple network with LSTM, both methods have an interesting training strategy. The gradient descent algorithm is used to compute the gradient step by step and hyper-parameters are updated with the loss on the testing set. However, this proposed method can perform well without updating parameters over several steps on new tasks. From Table 1, the result is 3.17% better than the MAML on 5 way-1 shot, while it also improved by 3.90% on 5 way-5 shot. Other baseline methods also perform poorly, such as Prototypical nets [9] and Matching nets [8]. These methods seem to learn an embedding space, and the distance between points is computed by artificial metrics. Both methods need high-quality representation for classes. In particular, the relation net [14] designed a classifier with hyper-parameters instead of artificial metrics, which avoided learning more redundant information. The results are shown in Fig 6, the C-SAM network improved 1.43% and 1.69% than relation net separately on 5 way-1 shot and 5 way-5 shot. The proposed method outperforms the selected methods except the A Simple Neural Attentive Meta-Learner [21] (SNAIL) on 5 way-5 shot. The accuracies of all models are averaged over 600 test episodes. We also visual different attention maps of different layers of this network on *mini*imageNet, as shown as Fig 7. The attention module pays more attention to detail features on the input images when the network is deeper.

**Table 1:** Average test set classification accuracy on *mini*imageNet.

Model	Fine-tuning	5 way-1 shot	5 way-5 shot
**MAML [6]**	Y	48.70%	63.11%
**Matching nets [8]**	N	43.56%	55.31%
**Prototypical nets [9]**	N	49.42%	68.20%
**Relation net [14]**	N	50.44%	65.32%
**SNAIL[21]**	N	**55.71%**	**68.88%**
**Meta Networks[16]**	N	49.21%	-
**LSTM[5]**	N	43.44%	60.60%
**SAB network**	N	52.04%	66.16%
**CAB network**	N	51.84%	66.30%
**C-SAM network**	N	51.87%	67.01%

**Fig 8:**
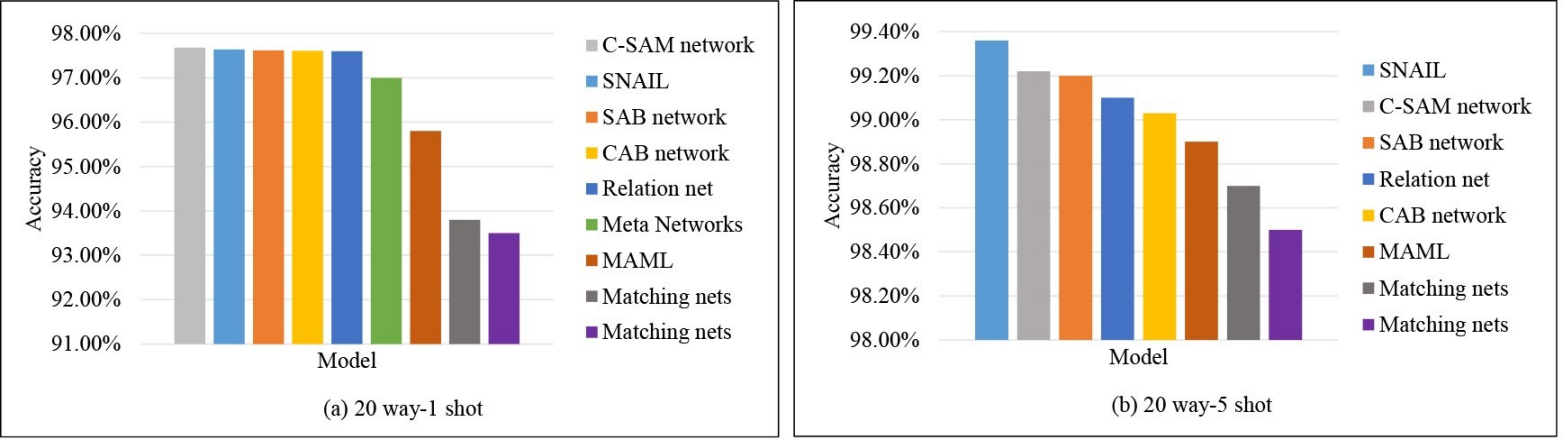
Accuracies sorted in descending order of each model for 20-way experiments on Omniglot.

As aforementioned in the analysis of the selected state-of-the-art models, we compare the C-SAM network with these state-of-the-art methods on Omniglot. The C-SAM network reaches 99.63% and 99.68% for both 5 way-1 shot and 20 way-1 shot separately, which is the best result of all listed models that are shown in Table 2, such as the Meta-learning with memory-augmented neural networks [31] (MANN), where the classification result reaches 82.8% and 94.9% for 5 way-1 shot and 5 way-5 shot. Although the MANN model stores new information with an external memory module using a number of read and write heads, it is unlikely to have enough memory to keep new information which is rapidly encoded. For 20 way-1 shot experiments, the C-SAM network outperforms the relation net and SNAIL from Fig 8.

**Table 2:** Average test set classification accuracy on Omniglot.

Model	Fine- tuning	5 way-1 shot	5 way-5 shot	20 way-1 shot	20 way-5 shot
**MAML[6]**	Y	98.7%	**99.9%**	95.8%	98.9%
**Matching nets [8]**	N	98.1%	98.9%	93.8%	98.5%
**Matching nets [8]**	Y	97.9%	98.7%	93.5%	98.7%
**Relation net [14]**	N	99.6%	99.8%	97.6%	99.1%
**SNAIL[21]**	N	99.07%	99.78%	97.64%	**99.36%**
**Meta Networks[15]**	N	99.0%	**-**	97.0%	**-**
**MANN[31]**	N	82.8%	94.9%	-	-
**SAB network**	N	99.53%	99.72%	97.62%	99.20%
**CAB network**	N	99.55%	99.71%	97.61%	99.03%
**C-SAM network**	N	**99.63%**	99.75%	**97.68%**	99.22%

Although there are subtle differences existing in fine-grained samples, the C-SAM network can extract distinction without a complex network structure. It can be seen from Table 3, for 1-shot experiments, the C-SAM network achieves 59.82%, which is 3% higher than relation net. For 5-shot experiments, this approach achieves about 2.45% higher than the relation net. From Table 3, the different attention components of the C-SAM module are used separately. However, for the 1-shot experiments and 5-shot experiments, the CAB network outperforms the C-SAM network. It shows that the channel attention can mine more fine-grained global information.

**Table 3:** Average test set classification accuracy on *Caltech-UCSD Birds*.

Model	5 way-1 shot	5 way-5 shot
**MAML[6]**	52.6%	66.5%
**LSTM[5]**	45.13%	63.92%
**Relation net[14]**	56.57%	68.88%
**SAB network**	57.83%	70.49%
**CAB network**	**61.29%**	**72.66%**
**C-SAM network**	59.82%	71.33%

#### Other novel datasets

This paper also makes some experiments on other datasets, those datasets are described as follows:

1. **Describable textures dataset (DTD).** The DTD is a texture database [32], which includes 47 classes and each class contains 400 textural images in the wild. There are a total of 5640 images.
2. **Stanford Dogs.** The Stanford Dogs [33] contains images of 120 breeds of dogs from around the world. Each class contains a different number of images, but each class has more than 100 images.
3. **Stanford Cars.** The Stanford Cars [34] includes 196 classes of cars, there are a total of 16185 images.

All datasets are divided into the training set and the testing set. We randomly select 10 classes in the DTD dataset as the testing set and 37classes as the training set. We randomly select 20 classes in other two datasets as the testing set and the rest as the training set. We conduct experiments on 5 way-1 shot and 5 way-5 shot respectively, the experimental results are shown as Table 4. Accuracies of all models are averaged over 300 test episodes. For 5-shot experiments on DTD, the C-SAM network achieves 56.12%, but the LSTM network achieves the best result, because texture features are more complicated and the forget gate determine optimal values to remember, while the C-SAM network loses temporal information.

**Table 4:** Average test set classification accuracy on other datasets.

Dataset		Proto nets[9]	LSTM[5]	Relation net[14]	SAB network	CAB network	C-SAM network
**DTD**	**5 way-1 shot**	37.99%	46.10%	47.48%	46.78%	46.79%	**47.81%**
	**5 way-5 shot**	50.75%	**58.85%**	56.95%	58.84%	58.15%	56.12%
**Stanford Dogs**	**5 way-1 shot**	41.81%	36.06%	44.25%	45.84%	**48.97%**	48.83%
	**5 way-5 shot**	56.05%	50.08%	59.09%	59.92%	60.70%	**61.32%**
**Stanford Cars**	**5 way-1 shot**	46.86%	30.18%	59.65%	60.31%	**62.57%**	60.83%
	**5 way-5 shot**	59.26%	53.93%	72.51%	72.48%	74.45%	**74.47%**

### Analysis of loss function and model generalization

#### Performance of different loss function

To improve the performance of the network, this paper also chooses two loss functions and evaluates them on *mini*imageNet. This dataset is used commonly in few-shot classification, and this proposed method also performs well on this dataset. For 5 way-5 shot experiments, we evaluate the SmoothL1Loss and BCELoss respectively. The SmoothL1Loss is less sensitive to abnormal feature, while the BCELoss can speed up the convergence of the network. The experimental results are shown as Table 5, the MSELoss performs well. The reason is that other loss functions require different features to meet some conditions, and this makes the important feature cannot be computed by the corresponding loss function.

**Table 5:** Average test set classification accuracy on different loss function.

Attention networks	MSELoss	SmoothL1Loss	BCELoss
**SAB network**	66.16%	65.83%	64.28%
**CAB network**	66.30%	**65.91%**	64.25%
**C-SAM network**	**67.01%**	65.87%	**66.55%**

#### Model generalization

To evaluate the capability of the model generalization. This paper conducts some experiments on other datasets that are mentioned in this paper using the trained model that is trained on *mini*imageNet, the experimental results are shown as Table 6. For both 5 way-5 shot and 5 way-1 shot experiments, the network achieves 54.99% and 39.78% on *CUB-200* respectively. However, the accuracies on other datasets are lower than the accuracies on *CUB-200*. This reason is that there are most different animal categories on *mini*imageNet, so that most related feature information is transferred to different categories existing in *CUB-200*. This indicated that prior knowledge is vital to classifier the new task.

**Table 6:** Generalization of the model on different datasets.

Dataset	5 way-1 shot	5 way-5 shot
**Stanford Dogs**	34.95%	47.93%
**Stanford Cars**	25.35%	31.56%
**DTD**	36.00%	50.41%
**CUB-200**	**39.78%**	**54.99%**

## Results and discussion

**Fig 9:**
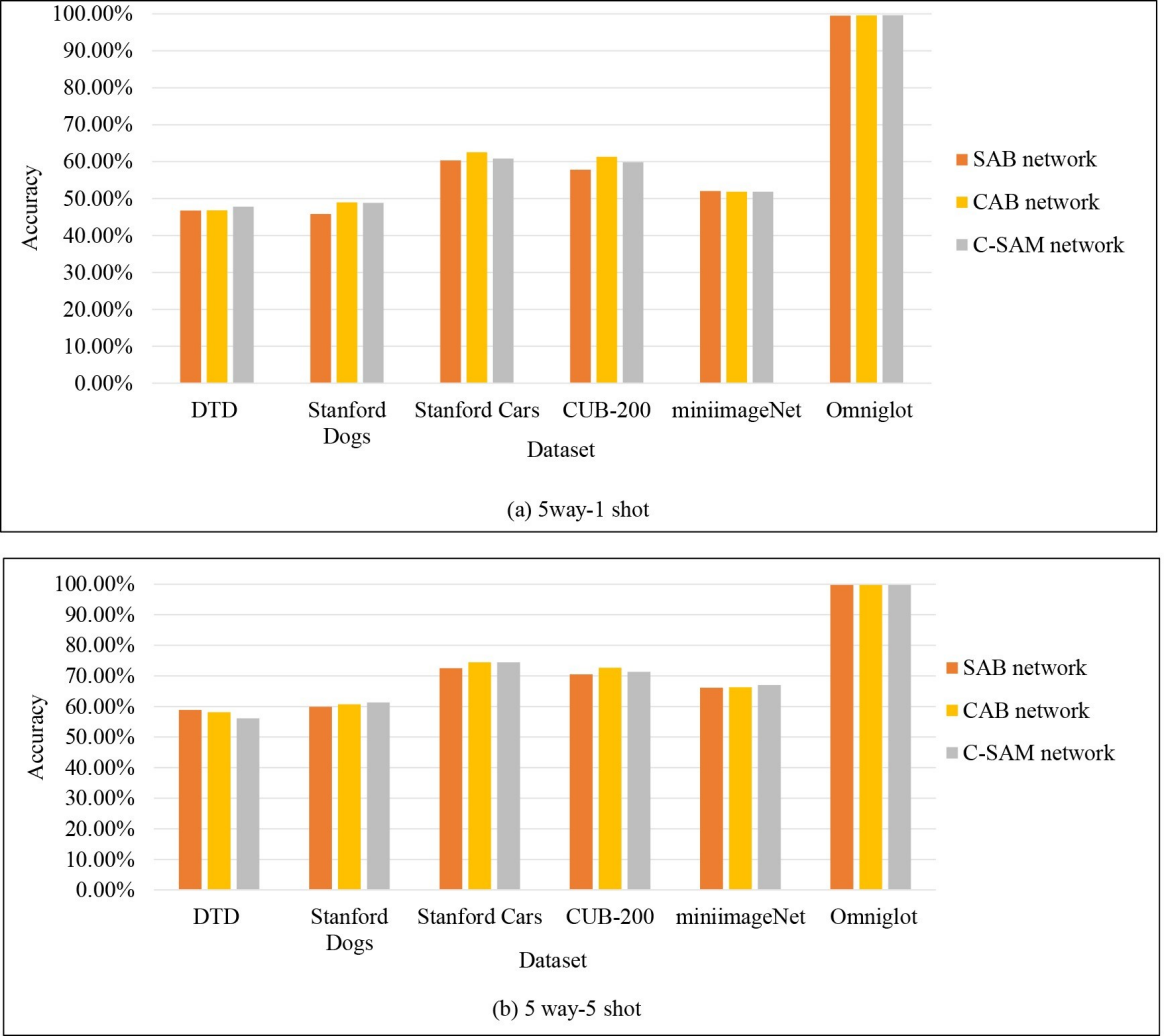
The contribution of different components on different datasets.

In the above experiments, the proposed method outperforms most state-of-the-art few-shot learning algorithms. For example, from the Table 1, although the SNAIL outperforms the C-SAM network on *mini*imageNet, the result is 0.56% and 0.04%lower than the result of this paper on the 5 way-1 shot and 20 way-1 shot experiments on Omniglot, because the SNAIL cannot obtain a great deal of information from the past sequence of the one labeled letters. Difference attention components of the proposed model are evaluated on different datasets, as shown as Fig 9. The separate attention network achieved well than listed state-of-the-art models, and there are different performances among those attention components on different datasets. For 1-shot experiments on *CUB-200* and novel datasets, the C-SAM network is worse than the CAB network, because there are only one training image with complex background, which contains few feature information. The spatial attention focuses on more redundant local information, while the channel attention focuses on global information that includes some detail information. If we combine spatial attention and the channel attention, the representation of the images of different tasks will contain more redundant information. On the contrary, the background of the images existing in tasks is simpler, the proposed model performs well. For example, the C-SAM network outperforms the SAB network and the CAB network on the Omniglot. For 5-shot experiments on novel datasets, the C-SAM network outperforms other branch networks, because the C-SAM network can obtain more powerful feature representation from several images instead of an image.

## Conclusion

In this paper, two popular attention mechanisms have been combined in the basic network. The channel attention mechanism focuses on which channel axis is important, and the spatial attention network finds that some important objects in each feature map. Both different types of attention mechanisms complement each other effectively to obtain more detailed information. Finally, the relation module is applied to compare the similarity between different samples. However, the proposed method has a shortcoming, which focuses only on extracting the detailed information and the temporal information is ignored. This makes that there are no relation between the training samples and training samples or testing samples. Therefore, the network can add LSTM to treat the entire task as a whole, so that each class is not independent. In addition, the samples existing in auxiliary tasks should have similar feature distribution as the samples existing in target tasks in feature space, which can generalize well on target task.
